# Efficacy and safety of tranexamic acid combined with low molecular weight heparin versus fondaparinux sodium following total knee arthroplasty: a retrospective cohort study

**DOI:** 10.3389/fphar.2025.1667528

**Published:** 2025-11-25

**Authors:** Abuduwupuer Haibier, Miying Pan, Dilxat Anwar, Pengcheng Ma

**Affiliations:** 1 Xinjiang Medical University, Xinjiang Uygur Autonomous Region, Urumqi, Xinjiang, China; 2 Huashan Hospital Fudan University, Shanghai, China; 3 Sixth People’s Hospital of Xinjiang Uygur Autonomous Region, Urumqi, Xinjiang, China; 4 Shandong Public Health Clinical Center, Shandong University, Jinan, Shandong, China

**Keywords:** osteoarthritis, total knee arthroplasty, low molecular weight heparin, fondaparinux, coagulation parameters, thrombosis

## Abstract

**Background:**

Low molecular weight heparin (LMWH) and fondaparinux (FPX) are commonly used to prevent deep vein thrombosis (DVT) following total knee arthroplasty (TKA). This study aimed to compare the efficacy and safety of tranexamic acid (TXA) combined with LMWH versus TXA combined with FPX in preventing DVT among TKA patients.

**Methods:**

A retrospective cohort study was conducted involving patients who underwent unilateral TKA at our institution between January 2020 and December 2023. Patients were divided into two groups based on their anticoagulation regimen: the TXA + LMWH group (n = 150) and the TXA + FPX group (n = 130). Perioperative indicators (blood loss, hospital stay, operative time, transfusion rate, transfusion volume, and total hospitalization costs), complications (DVT, muscular calf vein thrombosis [MCVT], surgical site infection, pulmonary thromboembolism, and postoperative hematoma), adverse reactions, coagulation parameters (D-dimer, prothrombin activity, INR, fibrinogen), and routine blood parameters (platelet count, hemoglobin, hematocrit) were compared between groups.

**Results:**

No significant differences were observed between groups in perioperative blood loss, operative time, hospital stay, transfusion rate, or volume (P > 0.05). While most preoperative baseline characteristics were comparable, the TXA + FPX group had significantly better baseline renal function (P < 0.05)0. On postoperative days 1 and 5, levels of D-dimer, prothrombin activity, INR, and fibrinogen were significantly lower in the TXA + LMWH group compared to the TXA + FPX group (P < 0.05). Total hospitalization costs were significantly lower in the TXA + LMWH group (P < 0.05). Additionally, the TXA + LMWH group exhibited a significantly lower overall complication rate (28.00% vs. 47.69%, P < 0.05) and lower incidence of MCVT (20.67% vs. 32.31%, P < 0.05). No significant differences were found in rates of DVT, surgical site infection, or postoperative hematoma (P > 0.05). No severe complications, such as pulmonary thromboembolism, acute renal failure, seizures, or death, occurred in either group.

**Conclusion:**

TXA combined with LMWH demonstrates significant advantages over TXA combined with FPX in reducing overall complications, particularly MCVT, and lowering hospitalization costs, with favorable improvements in coagulation parameters. Both regimens showed comparable efficacy in managing perioperative blood loss, operative time, hospital stay, and transfusion requirements in TKA patients. Given the retrospective design and limited sample size, further validation through high-quality, large-scale prospective studies is warranted.

## Introduction

Total knee arthroplasty (TKA) is a highly effective treatment for end-stage knee osteoarthritis, substantially improving patients’ quality of life. However, the procedure is associated with significant perioperative blood loss and an increased risk of venous thromboembolism (VTE), particularly DVT (Zheng and Qiu, 2017; Ning, 2021). These complications remain major concerns affecting postoperative recovery.

TXA, an antifibrinolytic agent, is now widely utilized for managing blood loss associated with TKA, effectively reducing transfusion requirements without substantially increasing thrombotic risk (Yao et al., 2018; Agarwal et al., 2014). Meanwhile, routine thromboprophylaxis with anticoagulants is essential for VTE prevention. Low-molecular-weight heparin (LMWH) and FPX are standard anticoagulants recommended by clinical guidelines (Bannuru et al., 2019; Katz et al., 2013). Fondaparinux (FPX), a selective factor Xa inhibitor, provides potent anticoagulation, while LMWH, with its broader inhibitory profile, remains widely used due to proven efficacy and cost-effectiveness (NIH, 2022).

The concomitant use of a hemostatic agent (TXA) and an anticoagulant presents a clinical challenge due to their seemingly opposing mechanisms. Although the safety and efficacy of TXA combined with various anticoagulants have been demonstrated, direct comparisons of TXA + LMWH versus TXA + FPX within the same patient population remain scarce (Kakar et al., 2009). Critical questions regarding their comparative impact on coagulation parameters, specific thrombotic complications (such as MCVT), and overall cost-effectiveness require further exploration.

Therefore, this retrospective cohort study aims to compare the clinical efficacy and safety of TXA combined with LMWH versus TXA combined with FPX in patients undergoing TKA, focusing specifically on perioperative blood loss, thrombotic and bleeding complications, coagulation profiles, and hospitalization costs.

## Materials and methods

### Study population

This study was conducted at the Department of Orthopedics, Our hospital between January 2020 and December 2023. Eligibility criteria were as follows:

Inclusion Criteria: ① Diagnosed osteoarthritis according to established criteria (Guidelines for the diagnosis and treatment of osteoarthritis, 2007) and age between 60 and 85 years; ② No previous history of TKA; ③ Complete clinical data and follow-up results available.

Exclusion Criteria: ① Revision surgery or bilateral TKA; ② American Society of Anesthesiologists (ASA) classification > Grade 3 (Hao et al., 2024); ③ Contraindications to LMWH or FPX, including allergy within the past 6 months or previous thrombotic events; ④ Preoperative anemia (hemoglobin <12 g/dL for females, <13 g/dL for males) (Xiong et al., 2023); ⑤ Body mass index (BMI) > 35 kg/m^2^; ⑥ Immunodeficiency or immune dysfunction; ⑦ Pregnancy or lactation; ⑧ Severe comorbidities.

#### LMWH group

Patients received a subcutaneous injection of 4,000 AXaIU, administered once daily at 4–6 h postoperatively.

#### *FPX* group

Patients received a subcutaneous injection of 2.5 mg, administered once daily beginning 6 h postoperatively.

A sequential thromboprophylactic regimen lasting 14 days was employed for all patients. This included initial in-hospital administration of LMWH or FPX, followed by oral rivaroxaban upon discharge. The choice of injectable anticoagulant during hospitalization was determined through shared decision-making between physicians and patients. The dosing regimen adhered to national expert consensus and recommendations from our hospital’s multidisciplinary team (Weber et al., 2017).

This study was approved by the Ethics Committee of the Sixth People’s Hospital of Xinjiang Uygur Autonomous Region, and written informed consent was obtained from all participants.

### Surgical procedures

All patients undergoing TKA underwent standardized preoperative assessments, including X-ray, CT, MRI, routine blood tests, liver and kidney function tests, and comprehensive physical examinations. All surgeries were conventional, cemented TKAs performed by the same surgical team, consisting exclusively of attending surgeons and anesthesiologists ranked associate senior attending or higher. A medial parapatellar surgical approach was uniformly employed, with anesthesia administered either generally or epidurally. A pneumatic tourniquet was consistently used at a pressure of 53.0 kPa, inflated prior to skin incision and released immediately after wound closure.All knee prostheses were cemented ceramic femoral component models from the ATTUNE® Knee System (Johnson & Johnson, Shanghai, China), coupled with cemented tibial trays. The tibial construct included offset tibial baseplates, femoral condyles, and locking lugs, which were fabricated using additive manufacturing technologies to optimize the osseointegative interface. Prior to joint capsule closure, a standardized periarticular injection (50 mL “cocktail mixture”) was administered uniformly, consisting of: 1 mg Compound Betamethasone Injection (Tianjin Kingyork Group Hubei Tianyao Pharmaceuticals Co., Ltd., Approval No. H42020019), 10 mL (20 mg) Ropivacaine (Yichang Humanwell Pharmaceutical Co., Ltd., Approval No. H20103552), and 0.3 mL (0.3 mg) Epinephrine Hydrochloride Injection (Grand Pharma [China] Co., Ltd., Approval No. H42021700), diluted with normal saline to a total volume of 50 mL. The TXA protocol (5 mL: 0.5 g, Yangtze River Pharmaceutical Group Nanjing Hailing Pharmaceutical Co., Ltd., Approval No. H20123005) included an intravenous infusion of 1 g TXA in 250 mL normal saline administered 30 min before tourniquet inflation, followed by topical application of another 1 g TXA in 250 mL normal saline via soaking after successful prosthesis implantation. The dosing regimen was established based on national expert consensus and recommendations from our hospital’s anesthesia team.

### Postoperative management

1. Within 2 h postoperatively, all patients routinely received an infusion of 5% Glucose Injection (500 mL: 25 g, Sichuan Kelun Pharmaceutical Co., Ltd.) combined with Sodium Lactate Ringer’s Injection (500 mL, Xinjiang Huashidan Pharmaceutical Co., Ltd.) for volume expansion and electrolyte balance maintenance. Patients were subsequently transferred to the post-anesthesia care unit (PACU) for a standard 2-h observation period before returning to the inpatient ward.
2. All patients received standardized postoperative care and thromboprophylaxis prior to ambulation. Mobilization and strength training commenced on postoperative day 1. After discharge, oral rivaroxaban (Xarelto, 10 mg, Bayer AG) was prescribed for 15 days to continue thromboprophylaxis. Doppler ultrasound was routinely performed to screen for DVT at discharge, at the 1-month follow-up, or upon clinical suspicion of DVT. Chest CT pulmonary angiography (CT-PA) was conducted to detect pulmonary embolism when clinically indicated.

Transfusion Protocol: Blood transfusion was indicated for patients with hemoglobin levels below 70 g/L or for those with hemoglobin levels between 70 and 100 g/L accompanied by symptomatic anemia (e.g., dizziness, palpitations, dyspnea, or reduced exercise tolerance).

### Outcomes

#### Baseline data

Baseline data, including age, gender, BMI, surgical site, type of surgery, ASA Physical Status Classification, Age-Adjusted Charlson Comorbidity Index (ACCI), comorbidities (hypertension, diabetes, coronary heart disease, neurological diseases, respiratory diseases, and metabolic disorders), and preoperative laboratory indicators, were collected from electronic medical records (EMRs).

*The ASA Physical Status Classification System* (Shivali and Thiagarajan, 2022), established by the American Society of Anesthesiologists (ASA), is a standardized preoperative health assessment tool designed to evaluate anesthetic risk by categorizing patients into six classes based on physiological status and surgical risk. Class I includes healthy patients, whereas Class II comprises patients with mild systemic disease without functional impairment; both classes generally tolerate anesthesia and surgery well. Class III encompasses patients with severe systemic disease causing functional limitations. Class IV includes patients with severe, life-threatening systemic disease resulting in significant impairment of daily living activities and elevated perioperative mortality risk. Class V represents moribund patients unlikely to survive without surgery, and Class VI denotes brain-dead organ donors.

*The ACCI* (Charlson et al., 1994) incorporates patient age into the original Charlson Index score. This practical assessment tool can be quickly administered during admission history-taking or derived from medical records, enabling comprehensive evaluation. It remains the most widely adopted comorbidity scoring system. The ACCI score is calculated by summing individual comorbidity weights and adjusting for age by adding one point per decade after age 40 (1 point for ages 50–59, 2 points for 60–69, 3 points for 70–79, and 4 points for ≥80 years). Details are provided in Table 1.

**TABLE 1:** Calculation method for the ACCI.

Weight	Comorbidities	
a Comorbidities and assigned weights		
*1*	· Myocardial infarction	· Chronic obstructive pulmonary disease
	· Congestive heart failure	· Connective tissue disease
	· Peripheral vascular disease	· Ulcer disease
	· Cerebrovascular disease	· Mild liver disease
	· Dementia	· Diabetes without end-organ damage
*2*	· Hemiplegia	· Moderate or severe renal disease
	· Diabetes with end-organ damage	· Any tumor, without metastasis
	· Leukemia	· Lymphoma
*3*	· Moderate or severe liver disease	
*6*	· Metastatic solid tumor	· Acquired immunodeficiency syndrome (AIDS)

Score	Age range
b Age score	
*0*	<50 years
*1*	50—59 years
*2*	60—69 years
*3*	70—79 years
*4*	≥80 years

Calculation Formula: Total ACCI, Score = Sum of Comorbidity Weights + Age Score.

### Primary outcomes

The primary outcomes included perioperative indicators (blood loss, length of hospital stay, operative time, blood transfusion rate, transfusion volume, and total hospitalization costs) and complication rates (DVT of the lower extremities, MCVT, surgical site infection, PTE, and postoperative hematoma).

*Total Blood Loss was Calculated using the Gross equation* (Forster and Stewart, 2016) *and Nadler equation* (Sun et al., 2019). Total blood volume (TBV) was calculated as: TBV = k_1_ × height (m)^3^ + k_2_ × weight (kg) + k_3_, with coefficients k_1_ = 0.3669, k_2_ = 0.03219, k_3_ = 0.6041 for males; and k_1_ = 0.3561, k_2_ = 0.03308, k_3_ = 0.1833 for females. Total blood loss was then calculated using: TBV × (preoperative hematocrit–lowest postoperative hematocrit)/average hematocrit. Visible blood loss was defined as the sum of intraoperative blood loss and postoperative drainage volume. Hidden blood loss was calculated as: total blood loss–visible blood loss + volume of transfused red blood cells.

*Diagnosis of Lower Extremity DVT*: Lower extremity DVT diagnosis followed the “Guidelines for the Diagnosis and Treatment of DVT (Third Edition).” If marked asymmetric edema of the lower limbs occurred or if the patient complained of unilateral lower limb swelling and pain, thigh circumference (10 cm above the midpoint of the patella) and calf circumference (10 cm below the midpoint of the patella) were measured bilaterally. A side-to-side circumference difference exceeding 2 cm indicated possible DVT, prompting measurement of serum D-dimer levels. A D-dimer level exceeding 0.5 mg/L raised suspicion of lower extremity DVT, necessitating further evaluation with deep venous ultrasonography. DVT was definitively diagnosed if ultrasonography revealed a solid echogenic filling defect within the deep venous lumen, accompanied by interruption of color Doppler flow signals.

*MCVT* (Schwarz et al., 2010): Thrombosis confined to the gastrocnemius or soleus venous plexus, without involvement of the central deep veins, confirmed by ultrasound.

*Incision infection* (Berríos-Torres et al., 2017): Infection was defined according to CDC guidelines as either a superficial infection (incision redness or purulence within 30 days postoperatively) or a deep infection (purulence in deep tissues, a positive pathogen culture, or occurrence within 90 days postoperatively).

*Postoperative hematoma* (Streubel et al., 2011): Required meeting two criteria: (1) ultrasound or MRI confirmation of a periarticular fluid collection or hematoma with a diameter ≥5 cm, and (2) a hemoglobin drop ≥2 g/dL or requirement for surgical intervention.

All events were adjudicated by an independent committee based on imaging and clinical data. Monitoring for DVT and PTE continued until postoperative day 90, whereas infections and hematomas were assessed until hospital discharge.

### Secondary outcomes

Secondary outcomes included the following postoperative laboratory parameters: D-dimer, prothrombin activity, international normalized ratio (INR), fibrinogen, platelet count, hemoglobin, and hematocrit.

### Statistical analysis

Statistical analyses were performed using SPSS version 26.0. Continuous variables are presented as mean ± standard deviation (x̄ ± s). Data normality was assessed using the Kolmogorov–Smirnov test and normal Q–Q plots. Intergroup comparisons for normally distributed data were conducted using the independent samples t-test. For non-normally distributed data, the Mann–Whitney U test was employed. Categorical variables were analyzed using the chi-square test or Fisher’s exact test, as appropriate. A two-sided P value <0.05 was considered statistically significant.

## Results

### Comparison of general data

**FIGURE 1:**
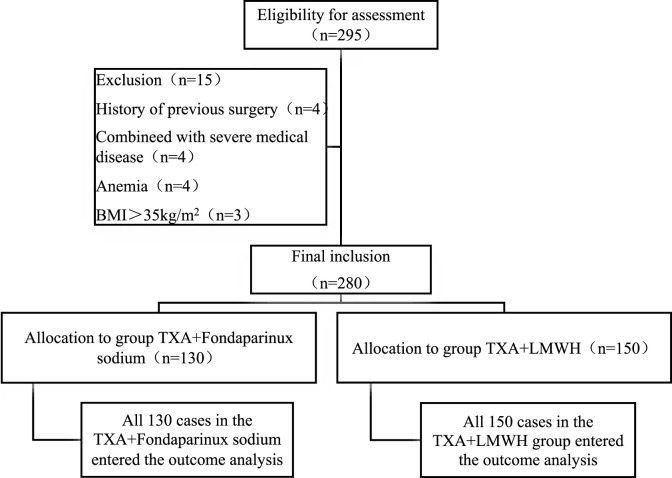
Flowchart of patient grouping.

A total of 280 patients undergoing TKA were allocated to the TXA + LMWH (n = 150) or TXA + FPX (n = 130) groups, with no dropouts (Figure 1). Baseline characteristics were comparable between groups except for renal function parameters, which were significantly better in the TXA + FPX group (P < 0.05; Table 2).

**TABLE 2:** Comparison of preoperative general data between groups.

*Index*	*TXA + LMWH (n = 150)*	*TXA +* FPX *(n = 130)*	*χ* ^*2*^ */Z/t*	*P*
Age (Years)	76.00 (72.00, 80.00)	76.00 (73.00, 79.25)	−0.110	0.913
Gender (n, male/female)	52/98	48/82	0.186	0.666
BMI(kg/m^2^)	23.50 (21.00, 26.00)	23.80 (21.00, 26.50)	−0.450	0.653
Preoperative blood glucose	7.612 ± 2.912	7.823 ± 3.312	0.532	0.596
Surgical site (left/right)	78/72	62/68	0.823	0.364
ACCI score (n)			0.581	0.901
0 ∼ 2	18 (12.00%)	15 (11.54%)		
3 ∼ 5	85 (56.67%)	75 (57.69%)		
6 ∼ 8	38 (25.33%)	32 (24.62%)		
≥9	9 (6.00%)	8 (6.15%)		
ASA Grade (n)			0.106	0.949
I	25 (16.67%)	23 (17.69%)		
II	100 (66.67%)	85 (65.38%)		
III ∼ IV	25 (16.67%)	22 (16.92%)		
Underlying disease (n)				
Hypertension	78 (52.000%)	62 (47.692%)	0.572	0.449
Diabetes	62 (41.333%)	48 (36.923%)	0.652	0.419
Circulatory system diseases	70 (46.667%)	58 (44.615%)	0.125	0.724
Neurological disorders	52 (34.667%)	32 (24.615%)	3.428	0.064
Respiratory diseases	28 (18.667%)	22 (16.923%)	0.164	0.685
Osteoporosis	54 (36.000%)	34 (26.154%)	3.286	0.070
Renal function				
Serum creatinine (μmol/L)	84.50 (72.00, 98.25)	78.00 (70.75, 88.00)	−2.350	0.019
eGFR (mL/min/1.73m^2^)	80.35 ± 15.62	85.92 ± 14.38	2.985	0.003
Complete blood count				
Platelets (10^12^/L)	208.50 (165.75, 248.25)	205.00 (155.00, 252.50)	−0.320	0.749
Hemoglobin (g/L)	115.00 (100.00, 130.00)	120.00 (105.00, 132.25)	−1.420	0.156
Hematocrit (%)	35.20 (31.30, 39.10)	35.90 (31.80, 40.00)	−0.870	0.384
Coagulation indices				
D-dimer (mg/L)	8.10 (6.60, 9.90)	8.70 (6.80, 10.90)	−1.650	0.099
PTA (%)	102.00 (92.00, 114.00)	103.50 (93.00, 117.25)	−0.710	0.478
INR	0.98 (0.90, 1.10)	1.01 (0.92, 1.15)	−1.210	0.226
Fibrinogen	3.50 (2.80, 4.30)	3.50 (2.80, 4.40)	−0.140	0.889

Data are presented as follows: continuous data as mean ± SD, or median (IQR); categorical data as n (%). Between-group comparisons were performed with

• Independent samples t-test: for normally distributed continuous data. • Mann-Whitney U test: for non-normally distributed continuous data. • Chi-square or Fisher’s exact test: for categorical data. • A two-sided p-value <0.05 was considered statistically significant. • INR, international normalized ratio; PTA, prothrombin activity; BMI, body mass index; eGFR, estimated glomerular filtration rate.

*Comparison of Perioperative Outcomes between groups:* No statistically significant differences were observed between the two groups in terms of perioperative blood loss, operative duration, hospital stay length, or transfusion requirements. However, total hospitalization costs were significantly lower in the TXA + LMWH group than in the TXA + FPX group (P < 0.05; Table 3).

**TABLE 3:** Comparison of perioperative outcomes between groups.

*Index*	*TXA + LMWH (n = 150)*	*TXA +* FPX *(n = 130)*	*χ* ^*2*^ */t*	*P*
Total blood loss (mL)	648.50 ± 807.80	706.25 ± 742.80	−0.623	0.534
Intraoperative blood loss (mL)	174.25 ± 126.40	178.50 ± 134.25	0.200	0.842
Length of hospital stay	16.85 ± 5.95	17.75 ± 9.35	0.183	0.855
Surgery duration	83.25 ± 25.85	86.15 ± 30.75	0.645	0.519
Total hospitalization cost (USD)	6,096.62 ± 1,931.07	6,689.76 ± 2,551.79	2.167	**0.031**
Transfusion rate	60 (40.00%)	66 (50.77%)	2.965	0.085
Blood transfusion volume	230.75 ± 352.80	298.50 ± 348.60	1.197	0.232

Data are presented as follows: continuous data as mean ± SD, or median (IQR); categorical data as n (%). Between-group comparisons were performed with.

• Independent samples t-test: for normally distributed continuous data. • Chi-square or Fisher’s exact test: for categorical data. • A two-sided p-value <0.05 was considered statistically significant.

**FIGURE 2:**
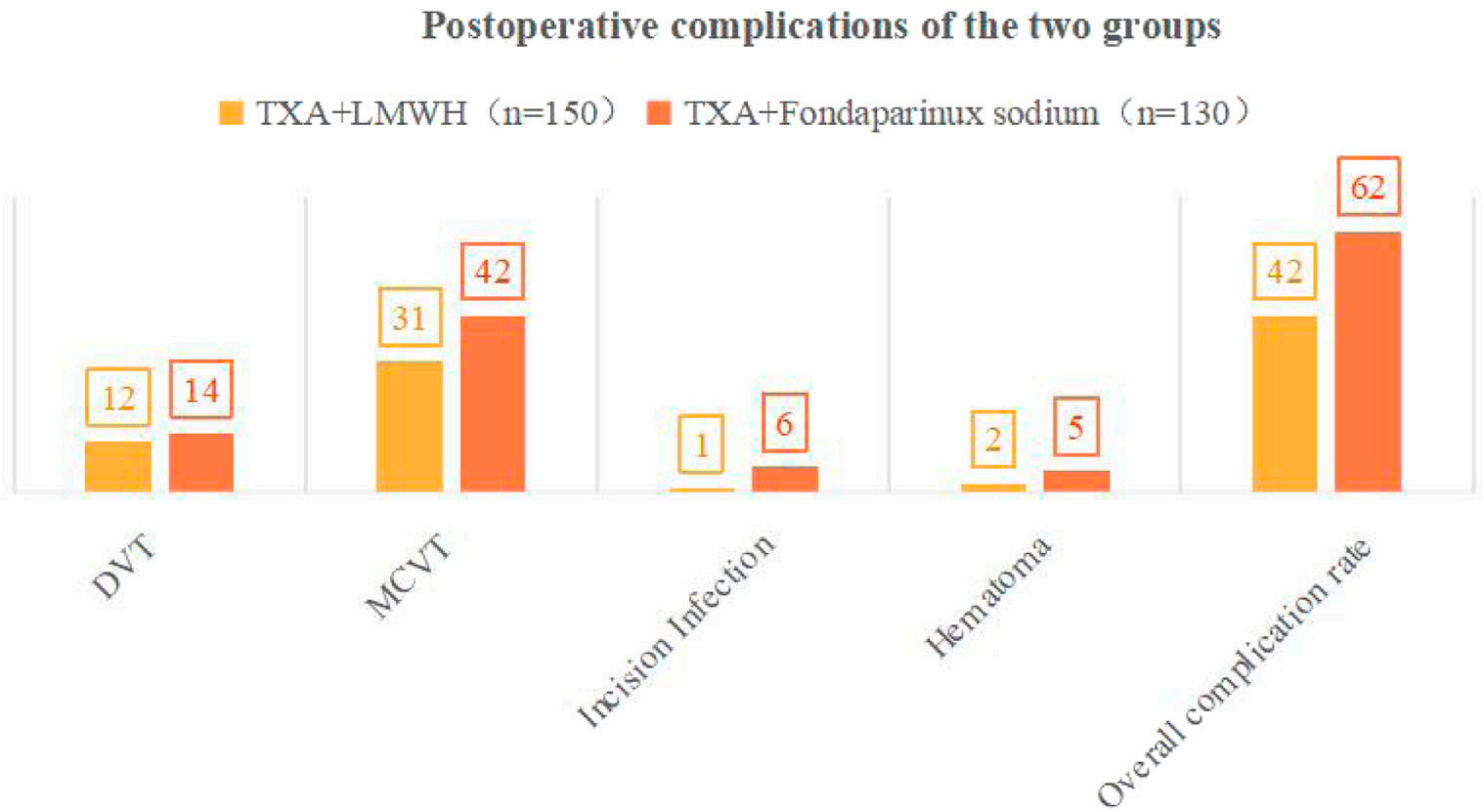
Postoperative complications between groups. Notes:·DVT, Deep Vein Thrombosis; MCVT, Muscular Calf Vein Thrombosis.

*Comparison of complications between groups:* The TXA + FPX group exhibited a significantly higher total complication rate (47.69%, 62/130) compared to the TXA + LMWH group (28.00%, 42/150; χ^2^ = 10.970, P < 0.05). Specifically, the incidence of MCVT was significantly higher in the TXA + FPX group (32.31% vs. 20.67%; χ^2^ = 4.620, P < 0.05). No statistically significant differences were observed between groups regarding DVT (10.77% vs. 8.00%; χ^2^ = 0.580), incision infection (4.62% vs. 0.67%), or hematoma (3.85% vs. 1.33%; all P > 0.05; Table 4; Figure 2). No severe complications, including pulmonary thromboembolism, acute renal failure, seizures, or death, occurred in either group, indicating comparable safety profiles for both anticoagulation regimens.

**TABLE 4:** Postoperative complications of the two groups.

*Index*	*TXA + LMWH (n = 150)*	*TXA +* FPX *(n = 130)*	x^2^	*P*
DVT (n)	12 (8.00%)	14 (10.77%)	0.58	0.447
MCVT (n)	31 (20.67%)	42 (32.31%)	4.62	0.032
Incision infection (n)	1 (0.67%)	6 (4.62%)	—	0.061*
Hematoma	2 (1.33%)	5 (3.85%)	—	0.252*
Overall complication rate (n)	42 (28.00%)	62 (47.69%)	10.97	**0.001**

• Data are presented as follows: categorical data as n (%). Between-group comparisons were performed with. • Chi-square or Fisher’s exact test: for categorical data, Items marked with an asterisk were analyzed using Fisher’s exact test*. • DVT: deep vein thrombosis; MCVT: muscular calf vein thrombosis. • Total complication rate = DVT + MCVT + Incision Infection + Hematoma. • No severe complications (pulmonary thromboembolism, acute renal failure, seizures, or death) occurred in either group.

Bold indicates statistically significant difference between the two groups (P < 0.05).

*Comparison of coagulation and routine blood parameters between groups on postoperative days 1 and 5*: On postoperative day 1, significantly higher levels of D-dimer, prothrombin activity (PTA), INR, and fibrinogen were observed in the TXA + FPX group compared to the TXA + LMWH group (P < 0.05). These differences persisted on day 5, with an additional significant difference noted in hemoglobin (all P < 0.05). No significant differences were detected in platelet count and hematocrit between the two groups at either time point (P > 0.05). Longitudinally, significant changes occurred from day 1 to day 5 in PTA, INR, platelet count, and hematocrit in both groups (all P < 0.05). However, hemoglobin levels did not change significantly in the TXA + FPX group (*P* > 0.05; Table 5).

**TABLE 5:** Comparison of coagulation indexes on day 1 and day 5 after surgery.

Index	Follow-up time	TXA + LMWH (n = 150)	TXA + FPX (n = 130)	*Z*	*P*
D-dimer (mg/L)	Day 1	3.20 (2.10, 4.85)	4.65 (3.40, 6.50)	3.271	0.001
	Day 5	3.50 (2.60, 5.10)	5.05 (3.70, 6.90)	3.912	<0.001
	*Z*	−0.841	−1.454		
	*P*	0.401	0.148		
PTA (%)	Day 1	96.00 (88.00, 108.00)	103.00 (92.00, 118.00)	3.643	<0.001
	Day 5	92.00 (86.00, 101.00)	95.00 (85.00, 112.00)	2.782	0.005
	*Z*	3.984	3.774		
	*P*	<0.001	<0.001		
INR	Day 1	1.12 (1.05, 1.20)	1.18 (1.10, 1.35)	3.688	<0.001
	Day 5	1.02 (0.95, 1.10)	1.12 (1.02, 1.28)	3.799	<0.001
	*Z*	5.090	2.094		
	*P*	<0.001	0.038		
Fibrinogen	Day 1	4.35 (3.70, 5.20)	5.05 (4.30, 6.10)	4.563	<0.001
	Day 5	4.60 (3.90, 5.50)	5.30 (4.50, 6.30)	4.149	<0.001
	*Z*	−2.205	−1.571		
	*P*	0.029	0.119		
Platelets (10¹²/L)	Day 1	235.00 (180.00, 315.00)	240.00 (185.00, 315.00)	0.165	0.869
	Day 5	265.00 (205.00, 350.00)	290.00 (220.00, 375.00)	1.400	0.162
	*Z*	−3.157	−4.641		
	*P*	0.002	<0.001		
Hemoglobin (g/L)	Day 1	98.00 (85.00, 115.00)	98.00 (87.00, 113.00)	0.124	0.901
	Day 5	95.00 (83.00, 110.00)	100.00 (86.00, 118.00)	2.194	0.028
	*Z*	1.955	−1.094		
	*P*	0.052	0.276		
Hematocrit (%)	Day 1	30.00 (27.00, 34.00)	30.50 (27.00, 35.00)	1.259	0.208
	Day 5	29.00 (26.00, 32.00)	29.50 (27.00, 33.00)	1.453	0.146
	*Z*	3.040	2.154		
	*P*	0.003	0.033		

Data are presented as follows: continuous data as mean ± SD, or median (IQR); categorical data as n (%). Between-group comparisons were performed with.

• Independent samples t-test: for normally distributed continuous data. • Independent samples t-test: for normally distributed continuous data. • Mann-Whitney U test: for non-normally distributed continuous data. • Chi-square or Fisher’s exact test: for categorical data. • A two-sided p-value <0.05 was considered statistically significant. • INR: international normalized ratio; PTA: prothrombin activity. • Normal reference ranges for laboratory parameters established by the institutional laboratory were as follows: D-dimer <0.5 mg/L (critical value for VTE, exclusion); PTA, 70%–130%; INR, 0.8–1.2; Fibrinogen 2.0–4.0 g/L; Platelets 125–350 × 10^12^/L; Hemoglobin 130–175 g/L (males) and 120–155 g/L (females); Hematocrit 40%–50% (males) and 36%–48% (females).

## Discussion

TXA effectively reduces blood loss and transfusion requirements in major orthopedic surgery. Evidence indicates that appropriate anticoagulation following TXA administration does not increase the risk of VTE (Weng et al., 2023; Zhao et al., 2014). Therefore, postoperative anticoagulation must balance efficacy and safety, with LMWH remaining the guideline-recommended option (Hill et al., 2010; Lin et al., 2011; Migliorini et al., 2024; Simon et al., 2023). *Consistent with previous findings, LMWH is the most frequently used anticoagulant alongside TXA following TKA, although warfarin and dabigatran etexilate are also common* (Tantavisut et al., 2025; Gillette et al., 2013). This retrospective cohort study provided a direct comparative analysis of TXA + LMWH versus TXA + FPX, addressing a notable gap in the literature. The novelty of our study lies in its integrated evaluation of clinical complications, coagulation profiles, and economic outcomes. While the two regimens showed similar results in perioperative blood loss, operative time, hospital stay, and transfusion rate (P > 0.05), the TXA + LMWH regimen was associated with significantly fewer overall complications (particularly MCVT), lower hospitalization costs (P < 0.05), and more favorable postoperative coagulation parameters (D-dimer, PTA, INR, and fibrinogen; P < 0.05). This multi-dimensional assessment suggests that TXA combined with LMWH may represent an optimized hemostatic--anticoagulant strategy following TKA.

FPX, a selective factor Xa inhibitor, produces a linear antithrombin-mediated effect and does not bind Platelet factor IV, thereby reducing the risk of over-anticoagulation and thrombocytopenia (Tantavisut et al., 2025; Gillette et al., 2013). Despite its stronger anticoagulant activity, our results show that FPX combined with TXA did not significantly increase total perioperative blood loss compared with LMWH (648.50 ± 807.80 mL vs. 706.25 ± 742.80 mL, P = 0.534). However, the FPX group exhibited higher rates of postoperative hematoma (3.85% vs. 1.33%) and surgical site infection (4.62% vs. 0.67%), along with a significantly higher overall complication rate (47.69% vs. 28.00%) and MCVT incidence (32.31% vs. 20.67%, P < 0.05). These observations are consistent with its pharmacological profile as a potent anticoagulant and align with meta-analyses reporting an increased risk of surgical site bleeding (OR = 1.43) (Kumar et al., 2019; Haibier et al., 2023; Smith et al., 2010; Fu et al., 2022; Mutlu et al., 2025).

Although FPX was associated with higher MCVT risk, no significant differences were observed in major safety outcomes between the two groups. Neither regimen caused severe complications, and bleeding risks remained well-controlled. This suggests that the standardized use of TXA effectively counteracted the inherent bleeding risks of both anticoagulants. Elevated postoperative coagulation activation markers (D-dimer and fibrinogen) in the FPX group indicate a possible disturbance in the coagulation–fibrinolysis balance, potentially increasing the risk of microcirculatory thrombosis (Kumar et al., 2019; Haibier et al., 2023; Smith et al., 2010; Fu et al., 2022; Mutlu et al., 2025; Karayiannis et al., 2022; Yang et al., 2021). Recent network meta-analyses further confirm that LMWHs maintain a more favorable bleeding profile than other anticoagulants, including FPX, in joint arthroplasty (Kumar et al., 2019).

The TXA + LMWH group also demonstrated significantly lower total hospitalization costs. Coupled with the reduced complication rate, LMWH showed superior cost-effectiveness, which is particularly relevant in resource-limited settings (Chen et al., 2022). These findings indicate that FPX may not be a cost-effective “gold standard” for post-TKA thromboprophylaxis when used with TXA. While meta-analyses support its efficacy in preventing proximal DVT, our data suggest that LMWH performs better in reducing distal MCVT. At our institution, the cost per dose of FPX (2.5 mg) is approximately $6.60 USD, compared with $2.70 USD for LMWH (enoxaparin 4000 IU) [exchange rate: 1 USD ≈7.25 CNY]. Although absolute costs vary across systems, FPX remains roughly 2.4 times more expensive, representing a key finding in cost-effectiveness analysis. Over a 2-week prophylactic course, this translates to substantial savings for the LMWH regimen. The higher incidence of MCVT in the FPX group may further increase costs due to additional imaging (e.g., Doppler ultrasound, ∼$16.50 USD per scan) and prolonged hospitalization. Thus, routine FPX use over LMWH is not economically justified in this context (Defrancesco et al., 2023; Huvers et al., 2005; Cheok et al., 2024). This multi-dimensional assessment suggests that TXA combined with LMWH may represent an optimized hemostatic–anticoagulant strategy following TKA. Our findings align with a previous study, which also reported that in patients undergoing joint replacement, compared to TXA combined with another anticoagulant, TXA + LMWH provided comparable clinical efficacy while demonstrating superior thromboprophylactic efficacy and lower hospitalization costs (Haibier et al., 2025).

However, an important consideration in interpreting these results is the potential for selection bias, inherent in our retrospective design. Fondaparinux is contraindicated in patients with severe renal impairment (creatinine clearance <30 mL/min) and requires dose adjustment or caution in moderate impairment, whereas LMWH has a broader therapeutic window (Ortel et al., 2020; Nutescu et al., 2009). In our cohort, the TXA + FPX group had significantly better baseline renal function, as evidenced by lower serum creatinine and higher estimated glomerular filtration rate (Table 2). This likely reflects clinical practice where physicians preferentially prescribe FPX to patients with preserved renal function. This systematic difference confounds the comparison: the group receiving the more potent anticoagulant (FPX) was also comprised of patients with inherently lower thrombotic risk due to better renal function. Consequently, the observed higher MCVT incidence in the TXA + FPX group might actually be an underestimation of its true risk relative to LMWH in a comparable patient population. This potential bias necessitates a more cautious interpretation of the complication rates and reinforces the conclusion that LMWH remains a robust and versatile option, particularly in unselected or renally impaired TKA populations.

This study was conducted in a unique geographic and demographic setting. The high altitude of Urumqi may predispose patients to elevated hematocrit and a hypercoagulable state, potentially increasing thrombotic risk such as MCVT. Moreover, the multi-ethnic composition of Xinjiang, including Uyghur, Han, and other groups, may contribute to genetic variability in thrombosis susceptibility and drug metabolism. These population-specific factors might have influenced the observed protective association of LMWH against MCVT and may limit generalizability to other regions or populations.

Several limitations should be acknowledged. First, the retrospective, single-center design and non-randomized anticoagulant allocation may introduce selection bias and limit generalizability. Second, the sequential thromboprophylactic regimen (in-hospital LMWH/FPX followed by post-discharge rivaroxaban) means that the outcomes reflect the entire prophylactic strategy rather than the in-hospital anticoagulant alone. This limits the direct attribution of effects, particularly beyond the hospitalization period, specifically to LMWH or FPX. Third, although the sample size was sufficient for primary outcomes, it was underpowered for detecting rare adverse events such as symptomatic pulmonary embolism. Fourth, laboratory evaluations were confined to postoperative days 1 and 5, providing only an early snapshot of coagulation status and missing potential later fluctuations or long-term normalization trends.In addition, the absence of anti-Xa monitoring prevented dose–response analysis, and the 90-day follow-up period was insufficient to assess long-term thrombotic risks.

In conclusion, this retrospective analysis suggests that for TKA patients, TXA combined with LMWH provides comparable perioperative hemostatic efficacy to TXA combined with FPX, while being associated with significantly lower complication rates, better coagulation recovery, and reduced hospitalization costs. The observed advantage of the LMWH regimen, particularly in reducing MCVT, may be even more pronounced given the better baseline renal profile of the FPX group. Although FPX remains an effective anticoagulant, our data indicate that LMWH with TXA may represent a more cost-effective and safer option in a broad clinical setting. Given the potential for selection bias and the unique demographic features of our cohort, these findings should be interpreted with caution, and their generalizability requires validation in prospective, randomized studies.

## Data Availability

The raw data supporting the conclusions of this article will be made available by the authors, without undue reservation.

## Abbreviations

TKA, Total knee arthroplasty; DVT, Deep vein thrombosis; TXA, Tranexamic acid; LMWH, Low-molecular-weight heparin; INR, International normalized ratio; MCVT, Muscular calf vein thrombosis; PTE, Pulmonary thromboembolism; ASA, American Society of Anesthesiologists; BMI, Body mass index; EMR, Electronic medical record; EBV, Estimated blood volume; PTA, Prothrombin activity; CDC, Centers for Disease Control and Prevention; CT, Computed tomography; MRI, Magnetic resonance imaging; PACU, Post-anesthesia care unit; CT-PA, CT pulmonary angiography.

## References

[B1] Agarwal S. Sharma R. K. Jain J. K. (2014). Periprosthetic fractures after total knee arthroplasty. J. Orthop. Surg. Hong. Kong 22 (1), 24–29. 10.1177/230949901402200108 24781608

[B2] Bannuru R. R. Osani M. C. Vaysbrot E. E. Arden N. K. Bennell K. Bierma-Zeinstra S. M. A. (2019). OARSI guidelines for the non-surgical management of knee, hip, and polyarticular osteoarthritis. Osteoarthr. Cartil. 27 (11), 1578–1589. 10.1016/j.joca.2019.06.011 31278997

[B3] BerríOS-Torres S. I. Umscheid C. A. Bratzler D. W. Leas B. Stone E. C. Kelz R. R. (2017). Centers for disease control and prevention guideline for the prevention of surgical site infection, 2017. JAMA Surg. 152 (8), 784–791. 10.1001/jamasurg.2017.0904 28467526

[B4] Charlson M. Szatrowski T. P. Peterson J. Gold J. (1994). Validation of a combined comorbidity index. J. Clin. Epidemiol. 47 (11), 1245–1251. 10.1016/0895-4356(94)90129-5 7722560

[B5] Chen L. Y. Khan N. Lindenbauer A. Nguyen T. H. (2022). When will fondaparinux induce thrombocytopenia? Bioconjug Chem. 33 (8), 1574–1583. 10.1021/acs.bioconjchem.2c00316 35878320 PMC9390334

[B6] Cheok T. Beveridge A. Berman M. Coia M. Campbell A. Tse T. T. S. (2024). Efficacy and safety of commonly used thromboprophylaxis agents following hip and knee arthroplasty. Bone Jt. J. 106-B (9), 924–934. 10.1302/0301-620X.106B9.BJJ-2023-1252.R2 39216864

[B7] Defrancesco C. J. Reichel J. F. Gbaje E. Popovic M. Freeman C. Wong M. (2023). Effectiveness of oral *versus* intravenous tranexamic acid in primary total hip and knee arthroplasty: a randomised, non-inferiority trial. Br. J. Anaesth. 130 (2), 234–241. 10.1016/j.bja.2022.11.003 36526484 PMC9900725

[B8] NIH (2022). Expert consensus on perioperative management strategies for accelerated recovery after hip and knee replacement in China. J. Chin. Orthop. Jt. Surg. 15(1):1–9.

[B9] Forster R. Stewart M. (2016). Anticoagulants (extended duration) for prevention of venous thromboembolism following total hip or knee replacement or hip fracture repair. Cochrane Database Syst. Rev. 3 (3), Cd004179. 10.1002/14651858.CD004179.pub2 27027384 PMC10332795

[B10] Fu D. Li L. Li Y. Liu X. Chen H. Wu N. (2022). Fondaparinux sodium and low molecular weight heparin for venous thromboembolism prophylaxis in Chinese patients with major orthopedic surgery or trauma: a real-world study. BMC Surg. 22 (1), 243. 10.1186/s12893-022-01652-6 35751113 PMC9229095

[B11] Gillette B. P. Desimone L. J. Trousdale R. T. Pagnano M. W. Sierra R. J. (2013). Low risk of thromboembolic complications with tranexamic acid after primary total hip and knee arthroplasty. Clin. Orthop. Relat. Res. 471 (1), 150–154. 10.1007/s11999-012-2488-z 22814857 PMC3528901

[B12] Guidelines for the diagnosis and treatment of osteoarthritis (2007). Chin. J. Orthop. Surg. 22 (03), 287–288.

[B13] Haibier A. Yusufu A. Lin H. Kayierhan A. Abudukelimu Y. Abudurexiti T. (2023). Efficacy and safety study of low-molecular-weight heparin and fondaparinux sodium after hip arthroplasty: a retrospective cohort study. Orthop. Res. Rev. 15, 253–261. 10.2147/ORR.S431372 38033454 PMC10684995

[B14] Haibier A. Aierxiding S. Yusufu A. Lin H. (2025). Efficacy and safety study of tranexamic acid combined with low-molecular-weight heparin and nadroparin calcium in postoperative application after joint replacement: a retrospective cohort study. BMC Musculoskelet. Disord. 26 (1), 826. 10.1186/s12891-025-08605-z 40890664 PMC12403312

[B15] Hao J. Pang P. Liu X. Chi W. Luo Z. Cai W. (2024). Can the lung ultrasound score predict pulmonary complications after non-thoracic surgery in patients with blunt thoracic trauma: a single-center observational study. J. Clin. Anesth. 99, 111675. 10.1016/j.jclinane.2024.111675 39504920

[B16] Harris R. N. Moskal J. T. Capps S. G. (2015). Does tranexamic acid reduce blood transfusion cost for primary total hip arthroplasty? A case-control study. J. Arthroplasty 30 (2), 192–195. 10.1016/j.arth.2014.08.020 25534861

[B17] Hill J. Treasure T. , and National Clinical Guideline Centre for Acute and Chronic Conditions (2010). Reducing the risk of venous thromboembolism in patients admitted to hospital: summary of NICE guidance. BMJ 340, c95. 10.1136/bmj.c95 20106897

[B18] Huvers F. Slappendel R. Benraad B. van Hellemondt G. van Kraaij M. (2005). Treatment of postoperative bleeding after fondaparinux with rFVIIa and tranexamic acid. Neth J. Med. 63 (5), 184–186. 15952489

[B19] Kakar P. N. Gupta N. Govil P. Shah V. (2009). Efficacy and safety of tranexamic acid in control of bleeding following TKR: a randomized clinical trial. Indian J. Anaesth. 53 (6), 667–671. 20640094 PMC2900076

[B20] Karayiannis P. N. Agus A. Bryce L. Hill J. C. Beverland D. (2022). Using tranexamic acid for an additional 24 hours postoperatively in hip and knee arthroplasty saves money: a cost analysis from the TRAC-24 randomized control trial. Bone Jt. Open 3 (7), 536–542. 10.1302/2633-1462.37.BJO-2021-0213.R1 35816170 PMC9350706

[B21] Katz J. N. Brophy R. H. Chaisson C. E. de Chaves L. Cole B. J. Dahm D. L. (2013). Surgery *versus* physical therapy for a meniscal tear and osteoarthritis. N. Engl. J. Med. 368 (18), 1675–1684. 10.1056/NEJMoa1301408 23506518 PMC3690119

[B22] Kumar A. Talwar A. Farley J. F. Muzumdar J. Schommer J. C. Balkrishnan R. (2019). Fondaparinux sodium compared with low-molecular-weight heparins for perioperative surgical thromboprophylaxis: a systematic review and meta-analysis. J. Am. Heart Assoc. 8 (17), e012184. 10.1161/JAHA.119.012184 31070069 PMC6585337

[B23] Lin P. C. Hsu C. H. Chen W. S. Wang J. W. (2011). Does tranexamic acid save blood in minimally invasive total knee arthroplasty? Clin. Orthop. Relat. Res. 469 (7), 1995–2002. 10.1007/s11999-011-1789-y 21286886 PMC3111781

[B24] Migliorini F. Maffulli N. Velaj E. Bell A. Kämmer D. Eschweiler J. (2024). Antithrombotic prophylaxis following total knee arthroplasty: a level I Bayesian network meta-analysis. Eur. J. Orthop. Surg. Traumatol. 34 (6), 2881–2890. 10.1007/s00590-024-04071-w 39126462

[B25] Mutlu T. Arican M. Karaduman Z. O. Turhan Y. Kaban İ. Dalaslan R. E. (2025). Effect of oral + topical and only topical tranaxamic acid application on blood loss and postoperative transfusion in primary total hip arthroplasty. J. Clin. Med. 14 (4), 1275. 10.3390/jcm14041275 40004805 PMC11856408

[B26] Ning J. Z. (2021). Main data of the seventh national population census. China Stat. (5), 4–5.

[B27] Nutescu E. A. Spinler S. A. Wittkowsky A. Dager W. E. (2009). Low-molecular-weight heparins in renal impairment and obesity: available evidence and clinical practice recommendations across medical and surgical settings. Ann. Pharmacother. 43 (6), 1064–1083. 10.1345/aph.1L194 19458109

[B28] Ortel T. L. Neumann I. Ageno W. Beyth R. Clark N. P. Cuker A. (2020). American society of hematology 2020 guidelines for management of venous thromboembolism: treatment of deep vein thrombosis and pulmonary embolism. Blood Adv. 4 (19), 4693–4738. 10.1182/bloodadvances.2020001830 33007077 PMC7556153

[B29] Schwarz T. Buschmann L. Beyer J. Halbritter K. Rastan A. Schellong S. (2010). Therapy of isolated calf muscle vein thrombosis: a randomized, controlled study. J. Vasc. Surg. 52 (5), 1246–1250. 10.1016/j.jvs.2010.05.094 20630682

[B30] Shivali S. Thiagarajan P. (2022). A practical guide to the American society of Anesthesiologists-physical status classification (ASA-PS). Indian J. Anaesth. 66 (4), 299–300. 10.4103/ija.ija_526_21 35663220 PMC9159401

[B31] Simon S. J. Patell R. Zwicker J. I. Kazi D. S. Hollenbeck B. L. (2023). Venous thromboembolism in total hip and total knee arthroplasty. JAMA Netw. Open 6 (12), e2345883. 10.1001/jamanetworkopen.2023.45883 38039005 PMC10692868

[B32] Smith S. B. Geske J. B. Maguire J. M. Zane N. A. Carter R. E. Morgenthaler T. I. (2010). Early anticoagulation is associated with reduced mortality for acute pulmonary embolism. Chest 137 (6), 1382–1390. 10.1378/chest.09-0959 20081101 PMC3021363

[B33] Streubel P. N. Ricci W. M. Wong A. Gardner M. J. (2011). Mortality after distal femur fractures in elderly patients. Clin. Orthop. Relat. Res. 469 (4), 1188–1196. 10.1007/s11999-010-1530-2 20830542 PMC3048257

[B34] Sun G. Wu J. Wang Q. Liang Q. Jia J. Cheng K. (2019). Factor Xa inhibitors and direct thrombin inhibitors *versus* low-molecular-weight heparin for thromboprophylaxis after total hip or total knee arthroplasty: a systematic review and meta-analysis. J. Arthroplasty 34 (4), 789–800. 10.1016/j.arth.2018.11.029 30685261

[B35] Tantavisut S. Artykbay S. Tangwiwat P. Susantitaphong P. (2025). Topical tranexamic acid in hip and knee surgery: a meta-analysis of randomized-controlled trials. EFORT Open Rev. 10 (7), 454–465. 10.1530/EOR-2024-0152 40591647 PMC12232384

[B36] Weber P. SteinbrüCK A. Paulus A. C. Woiczinski M. Schmidutz F. Fottner A. (2017). Partial exchange in total hip arthroplasty: what can we combine. Orthopade 46 (2), 142–147. 10.1007/s00132-016-3380-4 28083683

[B37] Weng N. Gou Y. Kuang F. (2023). Efficacy and safety of tranexamic acid in unicompartmental knee arthroplasty: a systematic review and meta-analysis. Asian J. Surg. 46 (8), 3033–3045. 10.1016/j.asjsur.2022.10.078 36396576

[B38] Xiong X. Li T. Cheng B. (2023). Anemia and formation of deep vein thrombosis before operation in patients with knee osteoarthritis: a cross-sectional study. J. Orthop. Surg. Res. 18 (1), 33. 10.1186/s13018-023-03518-w 36631873 PMC9835343

[B39] Yang Y. Wang Z. Wang F. Zhao X. Yang K. He J. (2021). Prospective, randomised, controlled study on the efficacy and safety of different strategies of tranexamic acid with total blood loss, blood transfusion rate and thrombogenic biomarkers in total knee arthroplasty: study protocol. BMJ Open 11 (2), e038399. 10.1136/bmjopen-2020-038399 33637540 PMC7919582

[B40] Yao M. H. Xu W. J. Shen W. Y. (2018). Application of rapid rehabilitation concept in single meniscus replacement patients. J. Qilu Nurs. 24 (20), 68–71.

[B41] Zhao Y. C. Yan G. Wei C. Yuejv L. Ying-Ze Z. (2014). Reduced blood loss after intra-articular tranexamic acid injection during total knee arthroplasty: a meta-analysis of the literature. Knee Surg. Sports Traumatol. Arthrosc. 22 (12), 3181–3190. 10.1007/s00167-013-2814-3 24352523

[B42] Zheng C. S. Qiu Z. Y. (2017). Analysis of the application value of ultrasound examination in early diagnosis of knee osteoarthritis. Intern. Med. 12 (5), 654–656.

